# Innovations in Skin and Soft Tissue Aging—A Systematic Literature Review and Market Analysis of Therapeutics and Associated Outcomes

**DOI:** 10.1007/s00266-023-03322-1

**Published:** 2023-05-08

**Authors:** Sumun Khetpal, Durga Ghosh, Jason Roostaeian

**Affiliations:** Division of Plastic and Reconstructive Surgery, David Geffen School of Medicine, University of California Los Angeles, 200 Medical Plaza, Suite 460, Los Angeles, CA 90095 USA

**Keywords:** Aging, Aesthetic surgery, Longevity economy, Biotechnology, Pharmaceuticals

## Abstract

**Purpose:**

Skin and soft tissue aging has been an important topic of discussion among plastic surgeons and their patients. While botulinum toxin, facial fillers, chemical peels, and surgical lifts preside as the mainstay of treatment to restore appearance of youth, emergent technologies, such as CRISPR-Cas9, proteostasis, flap biology, and stem cell therapies, have gained traction in addressing the aging process of skin and soft tissue. Several studies have introduced these advancements, but it remains unclear how safe and effective these therapeutics are in facial rejuvenation, and how they may fit in the existent treatment workflow for soft tissue aging.

**Materials/Methods:**

A systematic literature review was conducted to identify and assess therapeutics utilized in addressing skin and soft tissue aging. Variables collected included year of publication, journal, article title, organization of study, patient sample, treatment modality, associated outcomes. In addition, we performed a market analysis of companies involved in promoting technologies and therapeutics within this space. PitchBook (Seattle, WA), a public market database, was utilized to classify companies, and record the amount of venture capital funding allocated to these categories.

**Results:**

Initial review yielded four hundred and two papers. Of these, thirty-five were extracted after applying inclusion and exclusion criteria. Though previous literature regards CRISPR-Cas9 technology as the most favorable anti-aging innovation, after reviewing the current literature, stem cell therapies utilizing recipient chimerism appeared to be the superior skin anti-aging technique when accounting for possible disadvantages of various techniques. The psychosocial and cosmetic outcomes from using cell therapy to modulate allograft survival and tolerance may confer more long-term proposed benefits than the technologies in CRISPR-Cas9, flap biology innovations, and autologous platelet-rich plasma use. Market analysis yielded a total of 87 companies, which promoted innovations in technology, biotechnology, biopharmaceuticals, cell-based therapies, and genetic therapy.

**Conclusion:**

This review provides physicians and patients with relevant, usable information regarding how therapeutics can impact treatment regimen for facial aesthetics and skin rejuvenation. Furthermore, the goal of this research is to elucidate the varying therapeutics to restore appearance of youth, present associated outcomes, and in doing so, present plastic surgeons and their colleagues with greater insight on the role of these therapeutics and technologies in clinical practice. Future studies can further assess the safety and efficacy of these innovations and discuss how these may fit within surgical plans among patients seeking rejuvenation procedures.

**Level of Evidence III:**

This journal requires that authors assign a level of evidence to each article. For a full description of these Evidence-Based Medicine ratings, please refer to the Table of Contents or the online Instructions to Authors www.springer.com/00266.

## Introduction

Skin and soft tissue aging has presided as an important topic of discussion among plastic surgeons and their patients. Cutaneous aging has been cited to occur via intrinsic and extrinsic processes. Moreover, intrinsic aging occurs due to decreased proliferation of cells including keratinocytes, fibroblasts, and melanocytes, via a process termed cellular senescence [1]. During such processes, degeneration of fibrous extracellular matrix components such as elastin, fibrillin, and collagen, and decreased in vascularity occur. Collagen fibrils become disorganized, fragmented, and reduced in number and diameter. Extrinsic aging is primarily driven by exposure to ultra-violet (UV) radiation, which impairs differentiation process of epidermal keratinocytes and promotes accumulation of abnormal elastic tissue within the dermis [2–4]. Moreover, multiple molecular mechanisms have been proposed to mitigate the processes of skin and soft tissue aging; these include genomic instability, telomere attrition, epigenetic alterations, loss of proteostasis, deregulated nutrient sensing, mitochondrial dysfunction, cellular senescence, stem cell exhaustion, and altered intercellular communication [1–6].

Plastic surgery as a specialty has incorporated both noninvasive interventions, such as botulinum toxin, soft tissue fillers, bio-stimulants, chemical peels, platelet-rich plasma (PRP), and lasers, as well as surgical procedures, such as fat grafting, threading lifts, face and neck lift, blepharoplasty, and rhinoplasty, in efforts to promote a more youthful appearance among their patients. However, the application of newer technologies, such as genetic therapies, flap biology, and stem cell-based treatments, has gained traction among venture capitalists; the longevity economy has fielded $7.6 billion in hopes to promote healthspan and restoration of youth within the ever-growing aging population. Moreover, these advancements are yet to be integrated within the practice of most plastic surgeons and dermatologists. In addition, it remains unclear how safe and effective these therapeutics are in skin rejuvenation, and how they may fit in the existent treatment workflow for skin and soft tissue aging.

The purpose of this study was multi-fold: 1) to conduct a systematic literature review of therapeutics and technologies used to address skin and soft tissue aging processes; 2) to perform a market and trend analysis of venture-backed companies operating in the aforementioned indications; 3) to understand the safety and efficacy profiles of these respective innovations; 4) to discuss the implications for plastic surgeons when considering to implement them into their clinical practice. We hypothesized that cosmetic and aesthetic supplements would be the most common offering marketed by companies, given lower production costs and thus, lower barrier to entry, while innovations in stem-cell, proteostasis, and genetic therapy would be fewer yet the most lucrative in acquiring funding.

## Methods

A systematic literature review was conducted with National Center for Biotechnology Information, Medline, ASPS Tracking Operations and Outcomes for Plastic Surgeons, Cochrane, Web of Science, Scopus, and PubMed databases to identify articles that discussed therapeutics to address skin and soft tissue aging. A combination of the following keywords was utilized to conduct the literature review: “anti-aging,” “facial aesthetics,” “soft tissue aging,” “facial ageing,” “aesthetics,” “innovation,” and “skin ageing.” Variables collected included year of publication, journal, article title, organization of study, patient sample, and treatment modality, and level of evidence. Articles were excluded if data were published before 2000, yielded non-significant findings, or discussed already established treatments to mitigate skin and soft tissue aging processes **(**Table 1**).** Articles were then categorized by the level of evidence as set forth by the American Society of Plastic Surgeons (ASPS). Any conflicts were resolved through discussion and full text review among D.G. and S.K. Of note, the search was conducted according to the Preferred Reporting Items for Systematic Reviews and Meta-Analyses (PRISMA) guidelines. No funding was required to conduct this review of the literature.

**Table 1:** Summary of studies that discuss innovations in skin and soft tissue aging

Article title	Journal	First author	Year of publication	Proposed innovation	Mechanism	Conclusion	Level of Evidence
Targeting Alternative Splicing for Reversal of Cellular Senescence in the Context of Aesthetic Aging.	Plastic and Reconstructive Surgery	Bramwell et al	2021	Alternative Splicing	Splicing regulation of cells responsible for extra-cellular matrix remodeling, restoration of collagen and elastin frameworks, and reversal of adipose tissue atrophy	Opportunity for moderators of alternative splicing into injectable fillers or topical creams may allow genuine rejuvenation of skin and soft tissue.	V
Can Platelet- Rich Plasma Be Used for Skin Rejuvenation? Evaluation of Effects of Platelet-Rich Plasma on Human Dermal Fibroblasts	Annals of Dermatology	Kim et al	2011	Platelet-Rich Plasma (PRP)	Higher levels of PRP conferred increases in type I collagen, MMP-1 protein, and mRNA in human dermal fibroblasts.	PRP promotes tissue remodeling in aged skin and may be used as adjuvant treatment to lasers for skin rejuvenation in cosmetic dermatology.	III
Injectable Tissue-Engineered Soft Tissue for Tissue Augmentation	Journal of Korean Medical Sciences	Han et al	2014	Hyaluronic acid (HA) with mesenchymal stem cells	Injected patients with HA filler mixed with autologous cultured fibroblasts or SVF cells	Fibroblasts and adipose-derived stromal vascular fraction (SVF) cells may promote longevity of ECM and may be used via injectable tissue-engineered soft tissue.	III
Renuvion/J-Plasma for Subdermal Skin Tightening Facial Contouring and Skin Rejuvenation of the Face and Neck	Facial Plastic Surgery Clinics of North America	Gentile et al	2019	Cold atmospheric plasma (CAP) devices	Technology induces a restructuring effect in collagen fibers via subdermal tissue contraction	Renuvion offers many advantages in performing surgeries, such as rhinophyma reduction, skin tightening and rejuvenation, microinvasive, and facial and neck rejuvenation	IV
Innovations in natural ingredients and their use in skin care.	Journal of Drugs and Dermatology	Fowler et al	2010	Natural ingredients	Use of natural remedies including licorice, green tea, aloe vera, turmeric, for facial rejuvenation	Additional research is needed to determine to confirm and elucidate the benefits of these ingredients in the prevention and management of skin disease.	V
The role of gene therapy in regenerative surgery: Updated insights.	Plastic and Reconstructive Surgery	Giatstidis et al	2013	Genetic therapy—modification via viral vectors	Adenovirus vectors to manipulate epidermal growth factor (EGF), fibroblast growth factor (FGF), insulin-like growth factor (IGF), keratinocyte growth factor (KFG), platelet-derived growth factor (PDGF), transforming growth factor-β (TGF-β), and vascular endothelial growth factor (VEGF)	Effectiveness of “mature” gene therapy-based reconstructive procedures could soon set valuable milestones in the treatment of an extensive number of diseases.	V
Cellular proteostasis decline in human senescence.	Proceedings of the National Academy of Sciences of the United States of America	Sabath et al	2020	Proteostasis	Proteostasis decline is intrinsic to human senescence. Using transcriptome-wide characterization of gene expression, splicing, and translation, there was a significant deterioration in the transcriptional activation of the heat shock response in stressed senescent cells.	Proteostasis collapse, the diminished ability to maintain protein homeostasis, has been established as a hallmark of nematode aging.	III
Premise and promise of mesenchymal stem cell-based therapies in clinical vascularized composite allotransplantation.	Current Opinions in Organ Transplantation	Schweizer et al	2015	Stem Cell Therapy	Recipient chimerism is the holy grail of immunotolerance and may be facilitated by mesenchymal stem cell therapies that provide prolonged immunomodulatory, restorative, and regenerative changes.	Achieving immune tolerance may be possible by genetically reprogramming donor vascularized composite allografting tissue before transplantation or using cell therapy to modulate allograft survival and tolerance.	V
Treating chronic wound infections with genetically modified free flaps	Plastic and Reconstructive Surgery	Ghali et al	2009	Flap Biology	Ex vivo modification of free flaps may deliver cancer treatment, promote bone healing, and treat chronic wounds or infections This may help increase angiogenesis and reepithelization.	Creating genetically modified flaps to enhance the recipient site to which they are transferred has also grown traction as a new anti-aging therapy and mechanism	III
Stem cells in plastic surgery: a review of current clinical and translational applications	Archives of Plastic Surgery	Salibian et al	2013	Stem Cell Therapy	ADSCs may be suitable for promoting repair of atrophic and photo-damaged skin. ADSC injections increase dermal thickness and collagen density in aged mice, and reduce wrinkles induced by UVB-irradiation	These promising outcomes are similar to the results of translational studies, though further elucidation of the mechanisms behind these effects is necessary prior to further applying these therapies.	V
The role of a shelf-ready, human-derived, soft tissue injectable adipose matrix for facial volume correction	Journal of Cosmetic Dermatology	Gold et al	2020	Allograft Adipose Matrix (AAM)	The injectable AAM is readily available and provides a regenerative framework for sustainable results.	AAM may offer an alternative to synthetic fillers and autologous fat implantation in the face without the cumbersome process of fat harvesting and processing.	III
Adipose-Derived Mesenchymal Stem Cells (AD-MSCs) against Ultraviolet (UV) Radiation Effects and the Skin Photoaging	Biomedicines	Gentile et al	2021	Stem Cell Therapy	Adipose-derived mesenchymal stem cells act against skin photoaging in vitro via activation of dermal fibroblast proliferation, antioxidant effect, and matrix metalloproteinases (MMPs) reduction.	Summary of the most recent in vitro, in vivo and ex vivo outcomes and developments on the effects of AD-MSCs and F-GRF against photoaging.	III
Human tissue-resident stem cells combined with hyaluronic acid gel provide fibrovascular-integrated soft tissue augmentation in a murine photoaged skin model	Plastic and Reconstructive Surgery	Altman et al	2010	Stem cell therapy with hyaluronic acid	Adipose tissue-derived stem cells and hyaluronic acid induced a significant increase in procollagen 1-alpha-2 mRNA expression compared with controls.	The combination of adipose tissue-derived stem cells and nonanimal stabilized hyaluronic acid holds promise as a tool with which to achieve lasting volume fill in reconstructive surgical soft tissue augmentation.	III
Nanofat-derived stem cells with platelet-rich fibrin improve facial contour remodeling and skin rejuvenation after autologous structural fat transplantation	Oncotarget	Wei et al	2017	Stem cell therapy with platelet-rich fibrin, in addition to fat grafting	Nanofat-derived stem cells (NFSCs) were isolated, mechanically emulsified, cultured, and characterized. Platelet-rich fibrin (PRF) enhanced proliferation and adipogenic differentiation of NFSCs *in vitro*.	Transplants that combine newly isolated nanofat, which has a rich stromal vascular fraction (SVF), with PRF and autologous structural fat granules may therefore be a safe, highly effective, and long-lasting method for remodeling facial contours and rejuvenating the skin.	III
Subcutaneous fat tissue engineering using autologous adipose-derived stem cells seeded onto a collagen scaffold	Plastic and Reconstructive Surgery	Lequeux et al	2012	Stem cell therapy with collagen scaffold	Porcine autologous adipose-derived stem cells were isolated and seeded onto a three-dimensional collagen scaffold, and cultured for 10 days.	The authors' data clearly show the efficacy of adipose-derived stem cells for soft tissue repair and skin aging because it induces a significant increase in the dermis thickness.	III
Micro-Autologous Fat Transplantation Combined With Platelet-Rich Plasma for Facial Filling and Regeneration: A Clinical Perspective in the Shadow of Evidence-Based Medicine	Journal of Craniofacial Surgery	Ozer et al	2019	Autologous fat grafting with PRP	Pre-operative and post-operative FACE-Q surveys among patients who received fat grafting and PRP	A combination of platelet-rich plasma and micro-fat grafting with soft harvesting and processing could be seen a good surgical technique to restore volume and enhance skin quality in facial soft tissue augmentation.	IV
PN-HPT®(Polynucleotides Highly Purified Technology) in facial middle third rejuvenation. Exploring the potential	Journal of Cosmetic Dermatology	Cavallini et al	2022	Polynucleotides Highly Purified Technology (PN-HPT® )	Polynucleotides Highly Purified Technology (PN-HPT®) demonstrated dermal hydration and elasticity as well as fibroblasts vitality and activity in series of three treatment sessions.	PN-HPT® candidate as a valuable option for facial middle third rejuvenation.	III
The effect of cultured autologous fibroblasts on longevity of cross-linked hyaluronic acid used as a filler	Aesthetic Surgery Journal	Solakoglu et al	2008	Autologous fibroblasts with hyaluronic acid	Autologous labeled cultured fibroblasts of the rats were injected intracutaneously alone and mixed with hyaluronic acid; skin biopsies taken	Cultured human dermal fibroblasts combined with hyaluronic acid can provide a suitable, biocompatible, and long-lasting material and should be regarded as a new method in dermal renovation even beyond their temporary filling effect.	II
The effect of oral collagen peptide supplementation on skin moisture and the dermal collagen network: evidence from an ex vivo model and randomized, placebo-controlled clinical trials	Journal of Cosmetic Dermatology	Asserin et al	2015	Oral collagen supplementation	Two placebo-controlled clinical trials performed to assess role of collagen peptides on skin hydration by corneometry, on collagen density by high-resolution ultrasound and on collagen fragmentation by reflectance confocal microscopy. Human skin explants were used to study extracellular matrix components in the presence of collagen peptides ex vivo.	Oral collagen peptide supplementation significantly increased skin hydration and collagen density; decreased the fragmentation of the dermal collagen network Ex vivo experiments demonstrated that collagen peptides induce collagen and glycosaminoglycan production.	I
Ingestion of bioactive collagen hydrolysates enhance facial skin moisture and elasticity and reduce facial aging signs in a randomized double-blind placebo-controlled clinical study	Journal of the Science of Food and Agriculture	Inoue et al	2016	Ingestion of bioactive collagen hydrolysates	Two randomized double-blind placebo-controlled clinical trial of ingestion of two types of collagen hydrolysates, which are composed of different amounts of the bioactive dipeptides Pro-Hyp and Hyp-Gly, and studied skin moisture, elasticity, wrinkles, and roughness, at baseline, and 4 and 8 weeks.	Use of the collagen hydrolysate with a higher content of Pro-Hyp and Hyp-Gly led to more improvement in facial skin conditions, including facial skin moisture, elasticity, wrinkles and roughness	I
Oral Hyaluronan Relieves Wrinkles and Improves Dry Skin: A 12-Week Double-Blinded, Placebo-Controlled Study	Nutrients	Hsu et al	2021	Oral hyaluronic acid (HA)	Placebo-controlled, randomized, double-blind trial of daily HA (120 mg) intake for 12 weeks in 40 healthy Asian men and women (aged 35-64 years); variables: evaluation of wrinkles, stratum corneum water content, the amount of transepidermal water loss, elasticity via image analysis.	Significant improvement in overall skin condition, indicating that oral ingestion of HA may suppress wrinkles and improve skin condition.	I
Ingestion of an Oral Hyaluronan Solution Improves Skin Hydration, Wrinkle Reduction, Elasticity, and Skin Roughness: Results of a Clinical Study	Journal of Evidence-Based Complementary Alternative Medicine	Gollner et al	2017	Oral HA	Studied effects of Regulatpro Hyaluron on skin moisture content, elasticity, skin roughness, and wrinkle depths in twenty female subjects with healthy skin 45-60 years of age.	Intake of the HA solution led to a significant increase in skin elasticity, skin hydration, and to a significant decrease in skin roughness and wrinkle depths	IV
Ingestion of BioCell Collagen(®), a novel hydrolyzed chicken sternal cartilage extract; enhanced blood microcirculation and reduced facial aging signs	Clinical Interventions in Aging	Schwartz et al	2012	Biocell Collagen (hydrolyzed chicken sternal cartilage extract)	Pilot open-label study, we of 26 healthy females who displayed visible signs of natural and photoaging in the face	that dietary supplementation with BCC elicits several physiological events which can be harnessed to counteract natural photoaging processes to reduce visible aging signs in the human face	IV
Autologous platelet concentrates for facial rejuvenation	Journal of Applied Oral Science	Buzalaf et al	2022	Autologous platelet derivatives including platelet-rich fibrin	Review of articles that discuss autologous platelet concentrates (APCs)	APC may induce greater collagen production relative to PRP but future clinical trials should be pursued to assess this	V
Platelet-rich plasma, the ultimate secret for youthful skin elixir and hair growth triggering.	Journal of Cosmetic Dermatology	Elghblawi et al	2018	Platelet-rich plasma (PRP)	Review of articles that discusses the various applications of PRP	Promising role of PRP in facial rejuvenation when combined with surgical procedures	V
Histologic Evidence of New Collagen Formulation Using Platelet-Rich Plasma in Skin Rejuvenation: A Prospective Controlled Clinical Study	Annals of Dermatology	Abuaf et al	2016	Platelet-rich plasma (PRP)	20 women underwent injection of PRP in right post auricular area and saline in the contralateral region	PRP promotes dermal collagen levels	II
Expanded Stem Cells, Stromal-Vascular Fraction, and Platelet-Rich Plasma Enriched Fat: Comparing Results of Different Facial Rejuvenation: Approaches in a Clinical Trial.	Aesthetic Surgery Journal	Rigotti et al	2016	Platelet-rich plasma (PRP), adipose stromal cells	13 patients underwent injection of SVF-enriched fat or expanded adipose-derived stem cells or fat plus PRP in the preauricular areas.	PRP did not have significant advantages in skin rejuvenation over the use of expanded adipose-derived stem cells or SVF-enriched fat.	II
A clinical study on the usefulness of autologous plasma filler in the treatment of nasolabial fold wrinkles.	Journal of Cosmetic Laser Therapy	Choi et al	2017	Autologous plasma filler	20 Korean patients with moderate to severe nasolabial wrinkles were treated with autologous plasma filler	Wrinkle assessment score and global aesthetic improvement scale improved with this intervention	III
A multicenter, double-blind, placebo-controlled trial of autologous fibroblast therapy for the treatment of nasolabial fold wrinkles	Dermatological Surgery	Smith et al	2012	Autologous fibroblasts	302 patients either treated with therapy or placebo, and were assessed using a validated wrinkle assessment scale	Autologous fibroblast is safe and effective for treatment of nasolabial wrinkles	III
Autologous cell therapy: will it replace dermal fillers?	Facial Plastic Surgery Clinics of North America	Weiss et al	2013	Autologous fibroblast therapy	Review of role and considerations for use of autologous fibroblast therapy in facial rejuvenation	Best suited for nasolabial wrinkle correction, not filler agent	V
A Prospective Multicenter Study to Evaluate the Safety and Efficacy of the Topical Application of MYOWNN, an Autologous Growth Factor Concentrate (AGFC) Serum, in Anti-Aging.	Cureus	Chandrashekar et al	2022	MYOWNN, autologous growth factor concentrate	Cohort used product and six parameters (spots, pores, wrinkles, texture moisture, pigmentation) were measured	Five months of application of MYOWNN™ serum significantly improved anti-aging and face rejuvenation and was also well-tolerated.	III
Characterization of the Tissue and Stromal Cell Components of Micro-Superficial Enhanced Fluid Fat Injection (Micro-SEFFI) for Facial Aging	Aesthetic Surgery Journal	Rossi et al	2020	Micro-superficial enhanced fluid fat injection (SEFFI)	Evaluated SEFFI samples via microscopy to assess fluidity and composition	Adipose stromal cells (ASCs) were isolated from processing, thus confirming the regenerative potential of SEFFI grafts	III
Facial autologous soft tissue contouring by adjunction of tissue cocktail injection (micrograft and minigraft mixture of dermis, fascia, and fat).	Plastic and Reconstructive Surgery	Erol et al	2000	Injectable mixture of dermis, muscle strips, fat tissue, and fascia for use in facial region	450 patients underwent application of tissue cocktail”	Improved take of graft over author’s use of fat-only grafts, stability over many years	III
String Fat/Dermis Graft for Correction of Wrinkles and Scars.	Plastic and Reconstructive Surgery	Guyuron et al	2019	Dermis or a combination of fat and dermis is harvested in the form of strings	39 patients underwent grafting to depressed facial scars and rhytids at 49 sites	String fat grafting is a simple and reliable method for addressing depressed scars and limited rhytids	V
Anti-aging treatment of the facial skin by fat graft and adipose-derived stem cells.	Plastic and Reconstructive Surgery	Charles-de-Sá et al	2015	Injection of fat graft with stromal vascular fraction	6 patients received injection in pre-auricular areas, and skin samples analyzed	Aforementioned injection promotes a skin rejuvenation effect	III

In addition, a formal analysis of Pitchbook (Seattle, WA), a platform used to provide private market data, was conducted in order to identify companies operating within the fields of aesthetic aging and longevity. Companies were isolated using the following search terms: “Longevity Biotech,” “VC-backed,” “Accelerator/Incubator-backed,” “Anti-Aging,” “Soft Tissue Aging,” “Aesthetic Surgery,” “Facial Aesthetics,” “Anti-Aging Skin,” “Autologous,” and “Longevity.” Companies were excluded from analysis if 1) not operating within the healthcare or life sciences sector, or 2) not addressing issues directly within skin and soft tissue aging processes. Companies were then classified into the following categories: personal products, biologic, platform technology, genetic therapy, and cell-based therapy. Company descriptions and aggregate funding amount to date were collected via respective company websites, Pitchbook, as well as Crunchbase (San Francisco, CA) [7].

## Results

### Literature Review

**Fig. 1:**
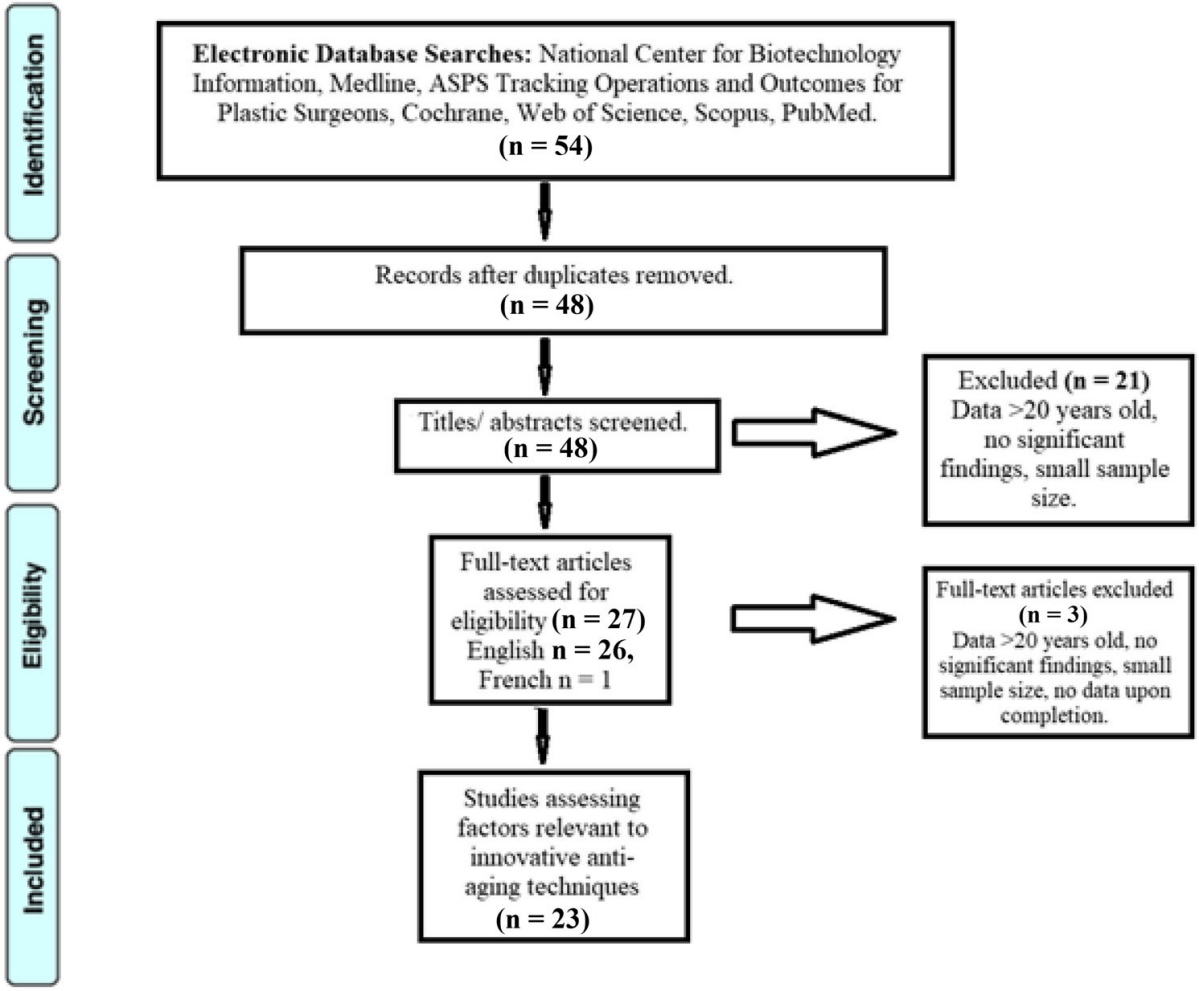
The PRISMA Flow Diagram for the systematic review detailing database searches, the number of abstracts screened, and the full texts retrieved

Initial review yielded a total of four hundred and two articles. After application of inclusion and exclusion criteria, a total of thirty-five studies were analyzed for the purposes of this publication. A flow-chart demonstrating the isolation of these studies is detailed in Fig. 1**.**

Genetic-based technologies, namely via maintaining cellular proteostasis, manipulating the process of alternative splicing for specific genes, and leveraging adenovirus vectors, were discussed in three studies. The majority of cited studies discussed stem cell-based innovations, specifically within adipose-derived mesenchymal stem cells (ADSCs), in combination with hyaluronic acid, fat grafting, PRP, and fibrin derived compounds, were additionally discussed as viable methods to address the sequela of photoaging and wrinkles in the aging process. Flap biology via genetic modification was also discussed as an option to mitigate the natural aging process within vascular composite allotransplantation, for example. Furthermore, oral collagen and hyaluronic acid (HA) supplements were also described in numerous studies. A summary of analyzed articles can be viewed in Table 1.

### Company-Level Analysis

**Fig. 2:**
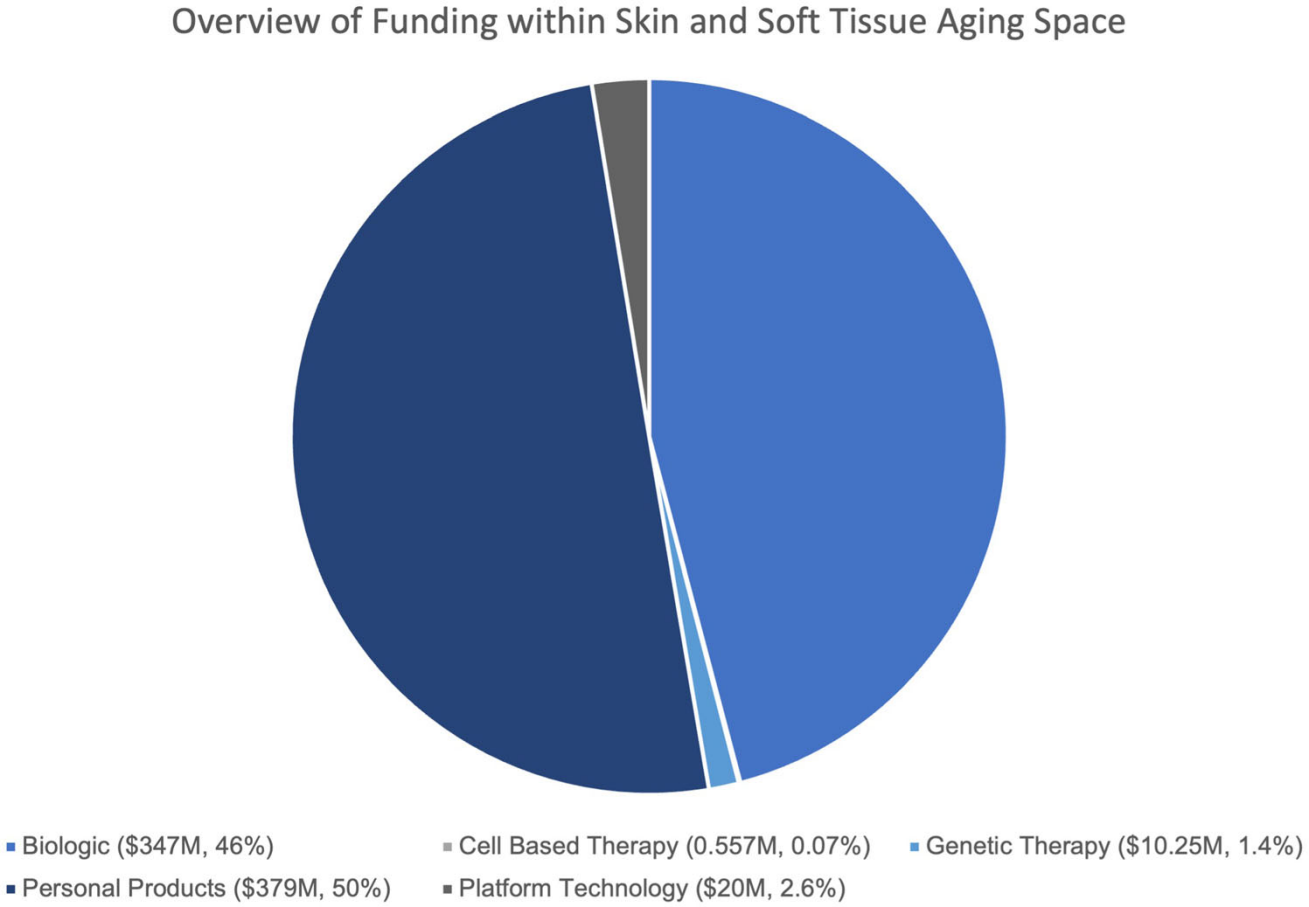
Overview of Funding within Skin and Soft Tissue Aging Space. Funding amount aggregates derived from Crunchbase and PitchBook data analytics

Initial search yielded a total of six hundred and seven companies, among which three hundred and fifty-seven did not operate within healthcare and the life sciences. The remaining two hundred and fifty companies were included in the analysis; these companies were classified into one of five categories, including platform technology, personal products, biologic, cell-based therapy, and genetic therapy. Our analysis demonstrated that the majority, 41% of companies hailed from the biologic space, followed by 24% from the genetic therapy space, 20% from personal products, 8% from cell-based therapies, and 7% from platform technologies. However, in terms of aggregated funding amount, personal products have gained the highest amount of funding, with an estimated $378,675,000 funding in the personal products space, closely followed by the biologic space with $347,531,000. The number of companies, as well as aggregated funding amount, for each category can be viewed in Fig. 2.

## Discussion

This study presents various mechanisms to address skin and soft tissue aging via literature and industry-level analyses. The literature review was largely comprised of articles that built upon already well-established methods for aesthetic rejuvenation, such as hyaluronic acid fillers, PRP, fat grafting, while the company-level analysis was dominated by personal products, such as supplements and skin care remedies.

### Cell-Based Therapies

The vast majority of articles included in the literature review alluded to the fundamental role that adipose-derived stem cells (ADSCs) have in tissue regeneration and remodeling processes. Moreover, in an analysis by Gentile et al, it was found that ADSCs directly activate dermal fibroblast proliferation, an antioxidant effect, and matrix metalloproteinases (MMPs) reduction [8, 9]. In combination with mainstay methods for facial rejuvenation, such as fat grafting, PRP, and soft tissue fillers, ADSCs have a multiplicative effect in refining skin texture and quality. Our market analysis drew attention to several companies building cell-based technologies, via exosomes as well as stem cells derived from adults and plants, to address skin and soft tissue aging [7, 10]. Future studies should further explore how ADSCs may be incorporated into the practice of plastic surgeons from a financial and logistical perspective [11].

### Autologous Methods

Our review of the literature also revealed multiple methods for leveraging use of autologous tissue to address skin and soft tissue aging. Moreover, several studies discuss the important role of platelet-rich plasma (PRP) within skin rejuvenation, through mechanisms of angiogenesis and mitogenesis [11–16]. Others have revealed opportunity for other autologous growth factors, fibroblasts, as well as combinations of dermis, fat, and fascia in the creation of various derivatives for facial rejuvenation [17–23]. Future investigations may explore long-term clinical outcomes, including safety and efficacy, associated with these respective therapeutics and additionally discuss the logistics entailed in utilizing these innovations in real clinical practice with patients.

### Genetic Therapies

Our investigation highlights the emergent role of genetic therapies, through multiple modalities, including alternative splicing, viral vectors, and epigenetics. Bramwell et al. leveraged the process of alternative splicing to regulate responsible for extra-cellular matrix remodeling, restoration of collagen and elastin frameworks, and reversal of adipose tissue atrophy [24]. In doing so, there exists an opportunity for moderators of alternative splicing to be incorporated into injectable fillers or topical creams for patients. Moreover, Giatstidis et al. proposed the notion of “mature” gene therapy, wherein adenovirus vectors can be utilized to manipulate epidermal growth factor (EGF), fibroblast growth factor (FGF), insulin-like growth factor (IGF), keratinocyte growth factor (KFG), platelet-derived growth factor (PDGF), transforming growth factor-β (TGF-β), and vascular endothelial growth factor (VEGF) [25]. Sabath et al. especially fixated on the role of genetic therapies preserving the process of cellular proteostasis [26–31]. Finally, companies, such as Ponce de Leon, have proposed that epigenetic modification (specifically DNA methylation) may be used to reverse aging processes [32–37].

### Biologics

Biologics, defined as small molecules targeting cellular pathways, have an established role in the anti-aging aesthetics industry. Furthermore, in the Pitchbook analysis, many companies, such as Dorian Therapeutics developed small molecules (senoblockers), selectively inhibit processes leading up to senescence, a process defined by cellular aging to the point of non-division [32–37]. Other companies developed molecules to directly target collagen scaffolds, ECM, and other cellular processes such as autophagy [38–44]. It remains unclear how these therapeutics compare to existent modalities for skin rejuvenation. Moreover, prior studies have yet to find definitive data as to where these therapeutics may preside in the treatment regimen proposed by plastic surgeons, dermatologists, and their colleagues.

### Personal Products and Natural Remedies

Natural ingredients, in the formulation of topical creams, lotions and preparations, have been used for anti-aging effects. Fowler et al describe ingredients, such as colloidal oatmeal, aloe vera, green tea, niacinamide and feverfew confer anti-inflammatory properties [45]. For hyperpigmentation and antioxidative capabilities, licorice, green tea, arbutin, soy, acai berry, turmeric and pomegranate have been described as efficacious. In our market analysis, multiple supplements that comprised of carotenoids, shark cartilage, plant derivatives have been taken by consumers for anti-aging effects [46–51]. In addition, collagen and hyaluronic acid (HA) supplements have conferred improvements in facial wrinkling and texture, as well as increases in skin elasticity and hydration [52–57]. Beyond aesthetic appearance, some supplements, namely those with nicotinamide, have been proposed to decrease risks of developing skin cancer [58]. Additional research is needed to elucidate and confirm the benefits of these ingredients in the management of skin and soft tissue aging, and possible skin cancer prevention.

### Flap Biology and Tissue Engineering

In a study done by Ghali et al, it was established that creating genetically modified flaps to enhance the recipient site may preside as a new anti-aging therapy and mechanism [59]. Researchers further state that CRISPR/Cas-9 technology would further facilitate concept by making this process more cost-effective, efficient, and multiplexed [60–63]. Moreover, this technology could be specifically applied to patients undergoing microsurgical reconstruction (head and neck, breast), and those undergoing vascular composite allotransplantation [10, 63–65]. The application of this innovation would ultimately refine aesthetic results in a way that promotes healthy skin quality and texture over time [66–69]. The precise application of genetically modified flaps allows engineering in anti-aging to be personalized and allow for limited scarring, proven time and time again to be of vital importance to patients [70–74]. Moreover, studies such as Dempsey et al have demonstrated genetically modified free flaps can be used without eliciting toxic systemic effects, through delivering IL-12 directly into the local environment of a skin tumor and suppress its growth [74]. The remarkable strides in research in flap biology and tissue engineering are currently being heavily applied to tumor suppression, but numerous studies have proved these effects would translate seamlessly to anti-aging applications [75–79]. Nonetheless, genetic modification of flaps may pose risks of increased rejection among patients. Future studies should explore these possibilities in pre-clinical studies to affirm the safety and efficacy of such ideas.

## Limitations

There are several limitations of this study that warrant consideration. First, the process of isolating and classifying companies within the Pitchbook database may be subject to inherent selection bias. In order to minimize such bias, the authors implemented standardized data collection forms, and additionally, consulted with one another in the event of a conflicting opinion. Second, our analysis was limited to companies within exclusively the Pitchbook database; thus, does not incorporate technologies from companies currently amid formation and raising capital. Third, while the investigation was able to elucidate the various methods toward addressing skin and soft tissue aging, measurement and comparison of outcomes (such as skin elasticity, dermal collagen density) were not incorporated within the analysis. Future studies may directly compare objective metrics among the cited therapeutics and technologies for the aging process. Despite these limitations, this study is the first to present a multi-level analysis, from academic and industry perspectives, to further explore the anti-aging landscape of innovation.

## Conclusions

Our study sought to explore the current modalities to address skin and soft tissue aging via literature and industry-level analyses. We concluded that there are multiple promising technologies and therapeutics within the anti-aging aesthetic industry, with innovations in genetic therapy, cell-based therapy, platform technologies, biologics, and personal products. The literature review was largely comprised of articles and journal pieces that built upon already well-established methods for aesthetic rejuvenation, such as hyaluronic acid fillers and fat grafting. In contrast, the company-level analysis was dominated by personal products, such as supplements and skin care remedies. In conducting this investigation, we present plastic surgeons and their colleagues with greater insight on the role of these therapeutics and technologies in clinical practice. Moreover, we provide investors relevant data on the growing industry of skin and soft tissue aging, detailing the most prominent categories of research. Future studies can further assess the safety and efficacy of these innovations and discuss how these may fit within surgical plans among patients seeking rejuvenation procedures.
